# Cockroach exposure and perceived stress interact to predict clinical outcomes in childhood asthma

**DOI:** 10.1186/s12890-021-01447-0

**Published:** 2021-03-12

**Authors:** Felicia A. Rabito, Derek Werthmann, Hua He, Aubrey S. Madkour, Whitney D. Arroyave, Michelle L. Sever, Thomas A. LaVeist

**Affiliations:** 1 Department of Epidemiology, School of Public Health and Tropical Medicine, Tulane University, 1440 Canal Street, Suite 2016, New Orleans, LA 70112 USA; 2 Department of Global Community Health and Behavioral Sciences, School of Public Health and Tropical Medicine, Tulane University, 1440 Canal Street, Suite 2016, New Orleans, LA 70112 USA; 3 PPD Government and Public Health Services , 3900 Paramount Parkway, Morrisville, NC 27560 USA; 4 Department of Health Policy and Management, School of Public Health and Tropical Medicine, Tulane University, 1440 Canal Street, Suite 2016, New Orleans, LA 70112 USA

**Keywords:** Childhood asthma, Cockroach allergen, Perceived stress, Path analysis, Asthma morbidity

## Abstract

**Background:**

Nonpharmacologic interventions for asthma management rely on identification and mitigation of important asthma triggers. Cockroach exposure is strongly associated with asthma morbidity. It is also associated with stress, another risk factor for asthma. Despite high prevalence of both in vulnerable populations, the impact of joint exposure has not been examined.

**Methods:**

Participants included 173 children with asthma in New Orleans, Louisiana. Cockroach exposure was based on visual inspection using standard protocols. Caregiver stress was measured using Cohen’s 4-item Perceived Stress Scale. Outcomes included unscheduled clinic or emergency department (ED) visits, hospitalization, and pulmonary function. Multivariable logistic regression was performed to assess independent effects of the exposure on the outcome and effect modification was examined in stratified analysis based on stress. Path analysis to explore the mediation effect by stress was performed using a probit link with parameters based on Bayes’ method with non-informative priors.

**Results:**

Adjusting for stress and other covariates, cockroach exposure was associated with unscheduled clinic/ED visits (aOR = 6.2; 95% CI 1.8, 21.7). Positive associations were also found for hospitalization and FEV_1_ < 80%. High stress modified the relationship with unscheduled clinic/ED visits (high aOR = 7.7 95% CI 1.0, 60.2, versus normal aOR = 4.1 95% CI 0.8, 21.9). Path models identified direct and indirect effects (*p* = 0.05) indicating that a majority of the total effect on unscheduled clinic/ED visits is attributed directly to cockroach exposure.

**Conclusion:**

The strong association between cockroach exposure and asthma morbidity is not due to uncontrolled confounding by stress. The combination of cockroach exposure and high stress, common in urban homes, are modifiable factors associated with poor asthma outcomes.

## Background

Asthma is the most prevalent chronic respiratory disease worldwide and is the leading chronic disease in children. In the United States (US), an estimated 6.2 million children have asthma [1]. Given that asthma typically starts in early childhood and is chronic in nature, poor asthma control has implications for pulmonary health throughout the life course.

Exposure to environmental allergens is a leading cause of asthma exacerbation, estimated to trigger asthma attacks in 60–90% of children [2] through promotion of airway inflammation and bronchial hyperresponsiveness [3]. Allergens commonly found indoors are particularly important due to increased exposure potential from time spent in the home. Many environmental allergens (e.g., house dust mites, cockroach, mold, pet dander, mice) are associated with asthma exacerbation, however studies around the world have found that exposure to cockroach antigen (e.g., Bla g 1, Per a 2) has the greatest effect, particularly on severe outcomes [4–7]. Among a cohort of moderate to severe asthmatics in New Orleans, Louisiana, children exposed to Bla g 1 > 2U/g were four times as likely to be hospitalized compared to their unexposed peers despite sensitization and exposure to multiple indoor allergens [5]. Similarly, in the National Cooperative Inner-City Asthma Study children in the US sensitized and exposed to cockroach were 3.4 times as likely to be hospitalized [6]. In Poland, 61% of children with cockroach sensitivity had severe asthma, compared to 36% of sensitized children exposed to other indoor allergens [8]. Among patients with persistent asthma in Taiwan, IgE-binding to American cockroach allergen (Per a 2) was associated with severe airway allergy and elevated proinflammatory chemokines [9].

Most studies of cockroach allergen and asthma outcomes have reported cockroach exposure based on measurement of allergens (e.g. Bla g 1) in dust samples from participants’ homes. However, visual inspection of homes, specifically looking for evidence of cockroaches has been shown to be predictive of allergen exposure. Cohn et al. reported that homes where field staff observed living or dead cockroaches or cockroach stains or occupants reported seeing cockroaches in the previous month were significantly more likely to have Bla g 1 allergen levels above 8 U/g [10].

Cockroach exposure is common, particularly in urban areas and in the home of those with low income status. Low-income households in the US have been found to be 12 times as likely to have high cockroach allergen (> 8 U/g) compared to households with high income [11–13]. Due to the high prevalence in inner city homes, research has primarily been conducted in urban cohorts. However, in addition to increased exposure to cockroach, inner city residence is also associated with high levels of stress, which is itself associated with negative asthma outcomes. Pertinent to childhood asthma, caregiver stress negatively impacts disease management[14] and can cause stress in the child leading to alterations in immune response (e.g., IgE, Bla g 2 proliferative response) and cytokine expression (e.g., IFN-γ, TNF-α, IL-10, IL-13) [15–17]. Exposure to stress has also been shown to increase susceptibility to environmental triggers by modulating the response to oxidative stress [18, 19]. Finally, poor housing condition, which is not uncommon in inner-city environments, is associated with cockroach infestation[20, 21] and has been shown in numerous studies to be linked to increased psychological stress [21, 22].

Despite the high prevalence of both cockroach exposure and stress in inner-city homes, to our knowledge there are no studies evaluating the impact of their joint exposure on childhood asthma morbidity. Therefore, it is possible that the strong association between cockroach and negative asthma outcomes may, in part, be the result of uncontrolled confounding by stress rather than the result of increased potency of cockroach antigen. Given that stress makes children more susceptible to asthma triggers, another possibility is that the strong role of cockroach may be the result of effect modification resulting from joint exposure [18, 23–25].

**Fig. 1:**
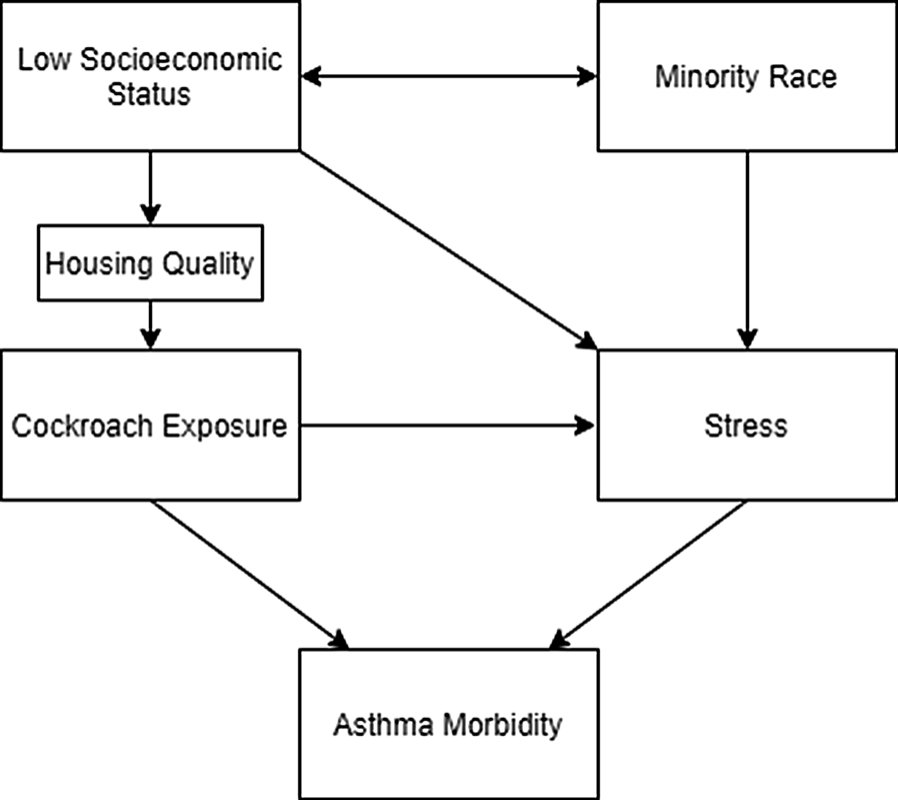
Biopsychosocial framework for caregiver stress, cockroach, and asthma

The objective of this analysis is to examine the relationship between cockroach exposure, stress, and asthma morbidity by evaluating various pathways by which they may be interrelated. The hypothesized relationships are rooted in the biopsychosocial framework (Fig. 1.) adapted from Wright et al. [26]. Based on this framework three hypotheses were tested. First, that previous findings on the unique association between cockroach exposure and asthma are biased by the lack of control for stress. Second, that joint exposure to cockroaches and stress results in effect measure modification. Finally, that cockroach impacts asthma through its association with stress [21, 26, 27]. To examine this, a mediation analysis examined the mechanistic process that underlies the relationship between cockroach, stress, and asthma.

## Methods

Data for this cross-sectional analysis were obtained from two asthma studies in New Orleans, Louisiana. Participants were children with parent-reported physician diagnosed asthma recruited between 2011 and 2015. Eligibility requirements included age 5–17 years, asthma-related symptoms in the previous 6 months or having at least two unscheduled clinic or emergency department (ED) visits for asthma within the past year. Additional eligibility criteria included the child sleeping in the home on average at least four nights per week in the preceding year and the caregiver’s ability to speak English or Spanish. Children with other severe medical or chronic illnesses, including chronic respiratory infections, were ineligible for the study. Children were recruited from asthma clinics and community outreach activities. Informed consent and assent for children seven years or older was obtained prior to data collection. The study received approval from the Tulane Biomedical Institutional Review Board.

### Health and environmental data collection

All data were collected in the child’s home. Sociodemographic characteristics were collected via survey questionnaire of the primary caregiver using a structured instrument administered by trained study personnel. Evidence of cockroaches in the home was dichotomized (yes/no) based on a home inspection by field staff using a protocol developed at North Carolina State University. Cockroach exposure was defined as observing active infestation, cockroach stains, excrement or other body parts, or dead cockroaches. This method has been used in previous studies and has been shown to be associated with antigen level and asthma morbidity [10, 28–30]. Caregiver stress was measured using Cohen’s 4-item Perceived Stress Scale (PSS-4). Stress was categorized as described by Kopel et al.; normal: PSS-4 score ≤ 7; high: PSS–4 score 8–10; and very high stress: PSS-4 score ≥ 11 [31]. High and very high stress were combined into one category and compared to caregivers with normal stress levels.

Asthma outcomes included parent reported ED or unscheduled clinic visits in the previous four months, parent reported hospitalization for asthma in the previous four months, and pulmonary function. Pulmonary function was measured via handheld spirometry (EasyOne™) using appropriate reference values and standard techniques [32]. Up to eight maneuvers were attempted to obtain a test of adequate quality. All results were reviewed to ensure a valid test was obtained. Children with FEV_1_ < 80% predicted were classified as having airway obstruction [33].

Covariates included the child’s age, parent-reported sex and race, smoking in the home, parent’s marital status, household income, and other household characteristics obtained from the caregiver via survey questionnaire. Height and weight were measured by study staff and body mass index (BMI) was calculated using the percentiles and z-scores (standard deviations) for the child’s sex and age based on the Centers for Disease Control and Prevention growth charts [34]. BMI was categorized in all analyses as follows: BMI < 5th percentile underweight; BMI ≥ 5th and < 85th percentile, normal weight; BMI ≥ 85th and < 95th percentile, overweight; and BMI ≥ 95th percentile, obese. A blood sample was obtained at the baseline home visit and analyzed using the ImmunoCAP specific IgE test (sIgE) for antibodies to German cockroaches (*Blatella germanica*), dust mites (*Dermatophagoides pteronyssinus* and *D. farinae*), cat dander, dog epithelium and dander, and mouse urinary proteins (Phadia, Uppsala, Sweden). Specific IgE level ≥ 0.35 kU/L was considered positive.

### Statistical analysis

Means and standard deviations are presented for continuous variables and number and proportion of participants are presented for categorical variables. Bivariate logistic regression models were constructed to examine independent associations and potential confounders: age (continuous variable), self reported sex at birth, self reported race (black versus other), BMI (underweight, normal, overweight, obese), smoker in the home (yes/no), and household income (≤ $25,000 verses > $25,000). Variables associated (*p* value ≤ 0.1) with asthma outcomes in bivariate analyses were included in multivariable logistic models. Both exposures, cockroaches and stress, were included in all multivariable models and outcomes were modeled separately. Adjusted odds ratios (aOR) with corresponding 95% confidence intervals (CI) are reported. To address whether stress level modifies the effect of cockroach exposure on asthma morbidity, we examined effect modification by caregiver stress level stratified as normal and high/very high. These analyses were performed using SAS statistical software version 9.4 (SAS Institute, Inc. Cary, North Carolina). Path analysis was performed to examine whether the effect of cockroach on the outcomes is mediated by level of caregiver stress and, if mediation exists, the extent of mediation. Unscheduled clinic/ED visits, hospitalizations and FEV_1_ < 80% were considered in separate path models. Cockroach exposure was considered the predictor variable and stress level the mediator. All three path models were adjusted for covariates identified to be associated in bivariate analysis for each corresponding outcome. The outcomes and mediator are binary variables, therefore a probit link was used in the path analysis and the parameters were estimated based on Bayes’ method with non-informative priors [35]. Indirect, direct, and total effects of cockroach exposure on asthma morbidity were computed based on the probit link functions for binary outcomes and mediators [36]. Goodness-of-fit was assessed using posterior predictive checking [37], and a posterior predictive p (PPP) value was obtained with a chi-square statistic measuring the distance between the data and the model. Larger PPP, i.e., smaller chi-square, indicates a better fit of the model to the data. Usually a model with PPP greater than 0.05 indicates a good fit [38, 39]. Mediation analysis was conducted using Mplus version 7 [40].

## Results

Table 1 describes the characteristics of 173 children included in the analysis. The mean age was 9.7 years. The population self-reported race was 70% non-Hispanic black and 26% Hispanic. The majority of families were headed by an unmarried adult, rented their homes, and had household income less than $25,000 per year. Most children were atopic (60%) and polysensitized (56%). Evidence of cockroaches was reported in 34% of homes and the mean caregiver PSS-score was 5.98 (SD 2.60). Approximately 40% of respondents reported high or very high stress. Of enrolled children, 49% had an unscheduled clinic or ED visit and 17% had been hospitalized for asthma in the previous 4 months while 47% had airflow obstruction (FEV_1_ < 80% predicted).

**Table 1:** Descriptive characteristics of the study population, n = 173

Variable	No	No. (%) or Mean ± SD
**Child’s Race/Ethnicity**	173	
Non-Hispanic Black		121 (69.9)
Hispanic		45 (26.0)
Other		7 (4.0)
**Household income**	166	
≤ $25,000		154 (92.8)
> $25,000		12 (7.2)
**Child’s age (years)**	173	9.7 ± 3.1
**Male Sex**	173	104 (60.5)
**Marital Status**	142	108 (75.5)
Single^a^		
**Home ownership**	155	136 (87.7)
Renter		
**Body Mass Index (percentile)**	139	
Underweight (BMI < 5th)		15 (10.8)
Normal weight (BMI ≥ 5th and < 85th)		67 (48.2)
Overweight (BMI ≥ 85th and < 95th)		13 (9.3)
Obese (BMI ≥ 95th)		44 (31.6)
**Smoker in Home**	142	46 (32.4)
**Cockroach Exposure**	136	45 (33.8)
**Perceived Stress Score (PSS-4)**	173	
Normal (PSS-4: ≤ 7)		104 (60.1)
High (PSS-4: 8–10)		65 (37.6)
Very high (PSS-4: ≥ 11)		4 (2.3)
**Sensitized** (sIgE ≥ 0.35 Ku/L)	151	
Cat		21 (14.2)
House Dust Mite		84 (56.8)
Dog		38 (25.7)
Mouse		16 (11.1)
Cockroach		40 (26.5)
≥ 1 allergen		91 (60.0)
≥ 2 allergens		84 (55.6)
**Health Care Utilization, previous 4 months**		
Unscheduled Clinic/ED Visit	173	86 (49.7)
Hospitalization	172	30 (17.4)
**Pulmonary Inflammation, FeNO**^**b**^	122	
> 20 ppb		32 (26.2)
≤ 20 ppb		90 (73.8)
**Pulmonary Function**	137	
FEV_1_% Predicted < 80%		65 (47.4)
FEV_1_% Predicted, Mean		85.39 ± 27.33

*Abbreviations*: ED, emergency department; FeNO, fraction of exhaled nitric oxide; FEV, forced expiratory volume; SD, Standard deviation

^a^ Raising child without a partner

^**b**^ Fractional Exhaled Nitric Oxide

In unadjusted models, (Table 2) cockroach exposure was associated with unscheduled clinic/ED visits (OR 8.74; 95% CI 3.87–19.71) and hospitalizations (OR 3.05; 95% CI 1.11–8.39). High caregiver stress was also associated with unscheduled clinic/ED visits (OR 2.88; 95% CI 1.53–5.42) and hospitalizations (OR 2.68; 95% CI 1.19- 6.0). Both cockroach exposure and high stress were positively associated with suboptimal lung function (FEV_1_ < 80%), but these associations were not statistically significant.

**Table 2:** Association between cockroach exposure, caregiver stress, and various indicators of asthma morbidity

Outcome	Cockroach				Stress			
	Unadjusted		Adjusted		Unadjusted		Adjusted	
	N	OR (95% CI)	N	OR^a^ (95% CI)	N	OR (95% CI)	N	OR^a^ (95% CI)
Unscheduled clinic/ED visits	136	8.7 (3.9, 19.7)	109	6.2 (1.8, 21.7)	173	2.9 (1.5, 5.4)	109	4.5 (1.4, 14.5)
Hospitalization	135	3.1 (1.1, 8.4)	130	2.3 (0.6, 7.8)	172	2.7 (1.2, 6.0)	130	5.6 (1.5, 21.0)
FEV1 < 80%	109	1.4 (0.6, 3.3)	108	1.8 (0.7, 4.8)	137	1.3 (0.7, 2.7)	108	1.2 (0.5, 2.7)

All models mutually adjusted for cockroach and PSS-score

^a^Control variables depend on results of bivariate analysis: Unscheduled clinic/ED visit: age, race, income, BMI; Hospitalization: sex, race, income; FEV_1_: age, BMI

Covariates significant in bivariate analysis, (age, race, income, BMI, sex) were included in multivariable models. In fully adjusted models (Table 2), cockroach exposure was positively associated with unscheduled clinic/ED visits (aOR 6.20, 95% CI 1.77, 21.73). Cockroach exposure was positively associated with hospitalizations (aOR 2.25, 95% CI 0.65, 7.81) and FEV_1_ < 80% (aOR 1.81, 95% CI 0.68, 4.85). Caregiver stress also remained significantly associated with unscheduled clinic/ED visits (aOR 4.50, 95% CI 1.40, 14.48) and with hospitalizations (aOR 5.60, 95% CI 1.49, 20.97).

To examine whether the relationship between cockroach exposure and asthma morbidity is modified by stress, multivariable models were stratified by stress level. Effect estimates differed substantially between those with high stress and those with normal stress. For all asthma outcomes, individuals exposed to both cockroaches and high stress had considerably higher odds of asthma morbidity (Table 3). Furthermore, when compared to fully adjusted, non-stratified models, effect estimates were noticeably higher for all outcomes. Children with caregivers reporting high stress and exposure to cockroaches had an aOR = 7.72 (95% CI 0.99, 60.22) for unscheduled clinic/ED visits while children with caregivers reporting normal stress and cockroach exposure, the aOR = 4.11 (95% CI 0.77, 21.87). For hospitalizations, subgroup differences were more pronounced. For children of caregivers with high stress, the aOR = 7.38 (95% CI 1.05, 51.85) while there was no association for children of caregivers reporting normal stress (aOR 0.26, 95% CI 0.04, 5.12). For pulmonary function (FEV_1_ < 80%) children with caregivers reporting high stress were 5.8 times as likely to have FEV_1_ < 80% (aOR 5.82, 95% CI 0.99, 33.80) while there was no association in those with normal stress levels aOR 0.74, 95% CI 0.19, 2.87.

**Table 3:** Association between cockroach exposure and asthma morbidity stratified by stress

Asthma outcome	High stress			Normal stress		
	N	aOR^a^ (95% CI)	*p* value	N	aOR^a^ (95% CI)	*p* value
Unscheduled clinic/ED visit	40	7.7 (1.0, 60.2)	0.05	69	4.1 (0.8, 21.9)	0.10
Hospitalizations	49	7.4 (1.1, 51.8)	0.04	81	0.3 (0.04,5.1)	0.34
FEV_1_ < 80%	39	5.80 (1.0,33.8)	0.05	69	0.7 (0.2,2.9)	0.67

*Abbreviations*: aOR, adjusted odds ratio; CI, confidence interval; ED, emergency department; FEV, forced expiratory volume

^a^Control variables: Unscheduled clinic/ED visit: age, race, income, BMI; Hospitalization: sex, race, income; FEV_1_: age, BMI

**Fig. 2:**
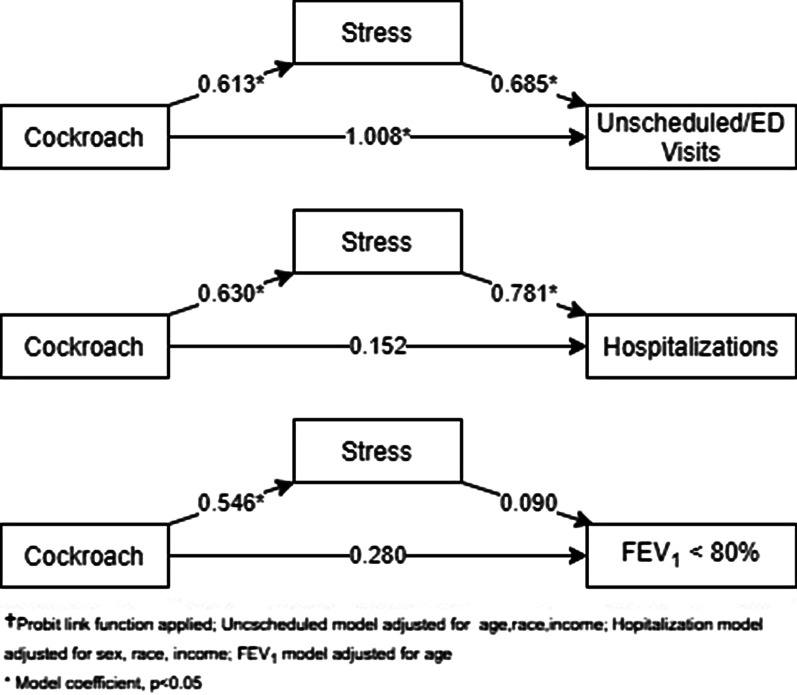
Path analysis of the mediating effect of stress on the relationship between cockroach exposure and asthma outcomes

Path analyses were conducted to examine whether the impact of cockroach exposure on asthma morbidity was mediated by stress level. The path models for each of the three outcomes assessed fit the data well. For unscheduled clinic/ED visits PPP = 0.480; for hospitalization PPP = 0.442; and for FEV_1_ < 80% PPP = 0.555. When examining the effect of cockroach exposure on unscheduled clinic/ED visits, the path model indicates that of the total estimated effects of cockroach exposure and stress, 91.1% of such effects are attributed to cockroach exposure, while 8.9% of such estimate effects are attributed to stress secondary to cockroach exposure (Fig. 2). As for the effect of cockroach exposure on hospitalization, 96.6% (95% CI: 55.2%, 131.0%) of the effect is attributed to cockroach exposure while 3.4% (95% CI: -31.1%, 44.7%) of the effect are attributed to stress secondary to cockroach exposure. The total and direct effect were significant, but the indirect effect was not significant. For FEV_1_ < 80%, 78.7% (95% CI −373%, 535.7%) of the effects are attributed to cockroach exposure while 21.3% (95% CI −438.8%, 465.2%) are attributed to stress secondary to exposure to cockroach.

### Discussion

Despite years of research dedicated to improving outcomes for children with asthma, our understanding of how the physical environment interacts with the social environment is limited. Research indicates a strong association between exposure to cockroaches in the home and asthma morbidity. The immune mechanisms underlying the relationship are unclear. One hypothesis is increased allergenicity of cockroach antigen, which induces an IgE response at concentrations 10–100 times lower than other allergens (assessed as nanograms of protein per gram of dust) [11–13, 41]. We hypothesized that the association seen in previous studies may be biased due to uncontrolled confounding by caregiver stress. This hypothesis was not substantiated. In mutally controlled and fully adjusted models there was a strong independent relationship for both cockroach exposure and caregiver stress on unscheduled clinic/ED visits and asthma hospitalization.

There is accumulating data on the interactive effects of environmental and psychosocial factors on children’s asthma. The biologic rationale is that both independently impact similar biologic processes (immune and non-immune inflammatory). Alterations of immune function and increased oxidative stress are posited to promote the synergistic effects of exposure on asthma [42]. These studies have addressed air pollution as the environmental contaminant. We have added to the growing body of literature by addressing another environmental factor, as recommended in a recent systematic review [43]on this subject. The stratified models examining whether the effect of caregiver stress amplifies the effect of cockroach exposure on asthma morbidity support this hypothesis; children jointly exposed to cockroach and high caregiver stress have greater likelihood of adverse asthma outcomes compared to children exposed only to cockroach. These findings are consistent with those of the systematic review that found psychosocial risk amplifies the relationship between air pollution, and various measures of asthma morbidity [43].

Finally, we addressed whether stress mediates the relationship between cockroach exposure and asthma. Results of path analysis show a marginal effect of cockroach on asthma morbidity mediated through stress and does not strongly support the hypothesis.

Findings from this analysis add to the growing body of evidence that social and environmental determinants of health interact to increase asthma morbidity and highlight the important role of cockroach exposure found in previous studies. Given the high prevalence of both caregiver stress and cockroaches in the home of inner-city children, the contribution of these factors to their asthma morbidity is likely substantial. Therefore, interventions to improve asthma outcomes are more likely to succeed if they target both cockroach exposure and stressors experienced by caregivers of children with asthma.

Several aspects of the study warrant consideration. We chose evidence of cockroaches as the exposure variable, a modifiable, patient-centered outcome. Although dust allergen levels are more often reported in research, the two are significantly correlated [10, 28–30]. To test the reliability between the estimates, we obtained dust samples for a subset of children in the study and compared the level of cockroach antigen to the categorization of exposure. For those categorized as having evidence of cockroaches, the median level was five times higher than those categorized as having no evidence of exposure. The mean for those with evidence of cockroach was 9.94 U/g compared to 1.64 U/g for those categorized as non-exposed which is below the threshold level of 2 U/g commonly used as the cut-point for the association with adverse health effects. The study has several limitations. The first is that use of evidence of cockroach rather than the amount of cockroach antigen in dust did not allow the examination of threshold and dose response effects. Although we included many covariates, we did not have information on air pollution. Another limitation is small sample size which limited the ability to fully interpret some results, particularly in stratified models. However, the results were consistent between models with the impact of small samples being generally less precise, yet strong positive effect estimates.

Strengths of the study include the inclusion of important covariates, namely BMI and smoking in the home. The use of evidence of exposure to cockroaches is a strength when considering the potential for translating the findings into practice. Behavioral interventions are unlikely to be adopted when they are difficult to implement or when the target population does not perceive a benefit. Evidence of cockroach is a patient-centered outcome while antigen level in dust is not.

## Conclusion

Identifying modifiable factors associated with asthma morbidity in children is a public health priority. This effort will benefit from models that consider a broad set of risk factors—both environmental and psychosocial—simultaneously. Our results confirm that both stress and cockroach are strong, independent risk factors for various measures of asthma morbidity. Our novel finding is that for children exposed to cockroaches with caregivers experiencing high stress, the effect of cockroach on asthma is significantly enhanced and the effect of cockroach on asthma is not mediated through its relationship with stress. This finding is important given the high prevalence of these factors in the homes of socially disadvantaged children living in urban environments. Future studies with larger sample sizes are needed. If the results are replicated, interventions reducing cockroach exposure should be emphasized, particularly in households where caregivers concurrently report high stress.

## Data Availability

The dataset used during the current study is available from the corresponding author on reasonable request.

## Abbreviations

ED: Emergency department
aOR: Adjusted odds ratio
CI: Confidence interval
FEV_1_: Forced expiratory value in one second
US: United States
Bla g 1: *Blattella germanica*
Per a 2: *Periplaneta americana*
IgE: Immunoglobulin E
IFN: Interferon
TNF α: Tumour necrosis factor alpha
IL-10: Interleukin 10
IL-13: Interleukin 13
PSS: Perceived Stress Scale
BMI: Body Mass Index
sIgE: Specific immunoglobulin E
PPP: Posterior predicted *p* value
OR: Odds ratio
SD: Standard deviation
